# Zirconolite Polytypes and Murataite Polysomes in Matrices for the REE—Actinide Fraction of HLW

**DOI:** 10.3390/ma15176091

**Published:** 2022-09-02

**Authors:** Sergey V. Yudintsev, Maximilian S. Nickolsky, Michael I. Ojovan, Olga I. Stefanovsky, Boris S. Nikonov, Amina S. Ulanova

**Affiliations:** 1 Institute of Geology of Ore Deposits, Petrography, Mineralogy and Geochemistry, Russian Academy of Sciences (IGEM RAS), 119017 Moscow, Russia; 2 A.N. Frumkin Institute of Physical Chemistry and Electrochemistry, Russian Academy of Sciences (IPCE RAS), 119071 Moscow, Russia

**Keywords:** actinides, immobilisation, matrix, zirconolite, polytype, murataite, polysome, scanning electron microscopy, electron backscatter diffraction

## Abstract

Electron backscatter diffraction (EBSD) has been used for more than 30 years for analyzing the structure of minerals and artificial substances. In recent times, EBSD has been widely applied for investigation of irradiated nuclear fuel and matrices for the immobilization of radioactive waste. The combination of EBSD and scanning electron microscopy (SEM/EDS) methods allows researchers to obtain simultaneously data on a specimen’s local composition and structure. The article discusses the abilities of SEM/EDS and EBSD techniques to identify zirconolite polytype modifications and members of the polysomatic murataite–pyrochlore series in polyphase ceramic matrices, with simulations of Pu (Th) and the REE-actinide fraction (Nd) of high-level radioactive waste.

## 1. Introduction

The development of sustainable nuclear power generation independent of uranium resources involves the reprocessing of spent nuclear fuel (SNF) with the recycling of uranium and actinides (Pu). The PUREX extraction process is industrially used for this, having initially been developed about 70 years ago in the USA to extract Pu and U for military purposes. Reprocessing a tonne of SNF generates 13–31 m^3^ of liquid high-level radioactive waste (HLW). HLW can contain stable and radioactive isotopes of fission products (Cs, Ba, I, Sr, REE, Mo, Zr, Tc, Ru, Rh, Pd), residual U and Pu, minor actinides (Np, Am, Cm), corrosion products (Zr, Ni, Cr, Mn, Fe, Co, Al), and technological impurities (Na, Fe, Al, S). Liquid HLW poses an environmental hazard and must be converted into a stable form for placement in a deep underground repository. Since 1978, HLW has been immobilised in B–Si glass (France, UK, USA, Belgium, etc.), and since 1987, in Al–P glass (Russia). About 30 thousand tonnes of borosilicate and almost 7 thousand tonnes of aluminophosphate vitrified HLW have been produced up to now, and this process continues. Significant volumes of solid and liquid HLW are stored at radiochemical plants in the USA and Russia.

The weak point of glass is the low HLW loading, which is 3–5 wt.% for Al–P and 15–20 wt.% for B–Si matrices, as up to 1.8 t (0.6 m^3^) Al–P and 0.4 t (0.15 m^3^) B–Si glass matrix is produced on processing of 1 t of spent nuclear fuel. This reduces the efficiency of underground disposal, including the search for locations and the construction process, which require considerable time and resources. Other drawbacks of glass includes potential decrease in retaining properties due to crystallization, and on contact with water the formation of radioactive colloids migrating in the geological environment. The problems of HLW management can be more effectively solved by partitioning HLW into groups of elements with similar properties, for their immobilization in optimal matrices. One of these groups is the fraction containing rare earth elements (REE) and minor actinides (MA) such as Am and Cm.

Russia is implementing a strategy of two-component nuclear power generation, with slow and fast neutron reactors operating in a closed nuclear fuel cycle [1]. This will reduce the need for uranium through the recycling of actinides, and will allow the extraction of useful stable and radioactive isotopes necessary for industry. The reprocessing of SNF will result in the generation of liquid high-level radioactive waste, so the development of methods for HLW optimal management is highly urgent. The greatest ecological concern is caused by long-lived actinides (Np, Pu, Am, Cm) and their daughter products [2,3,4], in particular, ^241^Am (T_½_ = 432 years) decays to form ^237^Np with a half-life of 2.1 million years.

In the advanced nuclear fuel cycle, MAs are extracted for transmutation in fast reactors in homogeneous (Np) and heterogeneous (Am) modes [1,4,5,6,7]. There is a proposal to store Curium for 70 to 200 years, to decay into Pu and fabricate nuclear fuel. Depending on the degree of actinide extraction, the radioactive waste hazard will be equal to the value for uranium ore in 300, 500, or 10,000 years [1,2,4,7,8,9]. This concept is known as “radiative equivalence”. In a shorter time, about 100 years, radiological (oncological) equivalence between them will occur [10]. The application of this approach requires the creation of sophisticated technologies for processing SNF and extracting transplutonium elements (TPE) from HLW. Methods for partitioning REE, americium, and curium with similar properties, the separation of Am and Cm, fabrication of fuel with Np and Am, and its processing after irradiation in a fast reactor are still far from being implemented [11,12,13,14]. The timing of closing the nuclear fuel cycle, involving the partitioning of minor actinides (Np, Am, Cm), fuel fabrication, its irradiation in fast reactors, and subsequent reprocessing, has been shifted to 2050 [15], which is more than 30 years longer than previous estimates.

Comparison of the potential harm to human health from radioactive waste and U ore (radiative and oncological equivalence) is based on the assumption of their complete dissolution in groundwater. However, there are no real grounds for this, since uranium deposits with an age of many millions of years are known, representing reserves of hundreds of thousands of tonnes at concentrations of up to 20 wt.%. Reliably established reserves of uranium in these deposits are approach six million tones, and estimated resources are 7.5 million tonnes. The number of known U deposits exceeds 1800, the oldest of which are over 2 billion years old [16,17,18]. Since the solubilities of HLW and uranium ores in groundwater are very low, it makes no sense to compare their hazards. Environmental hazard assessment through the volume of water required to dissolve elements to a safe level has shown that Hg, Se, Pb, Cd, and As ores containing the first wt.% of these elements pose an even greater environmental threat [19,20,21]. Unlike radionuclides, which gradually decrease in quantity due to decay, the danger from these toxic elements does not decrease with time.

The main industrial minerals of U are uranium IV compounds; oxides (uraninite), phosphate (ningyoite), silicate (coffinite), and titanate (brannerite). Their ores are mined by underground leaching with the use of oxidizers, acidic, or alkaline solutions. The stability of U, Th, and REE minerals such as pyrochlore, perovskite, zircon, monazite, and britholite is even higher [22,23,24,25]. Zirconolite is highly stable in nature, with isotope systems that have been closed for hundreds of million years, making it possible to use zirconolite for age determination [26]. Therefore, alternatives to transmutation methods for management of HLW actinide fraction are based on the existence of geological settings in which actinide migration is practically absent. This approach consists of actinide immobilization in stable matrices and their placement at depths from hundreds of meters to 3–5 km in mined or borehole disposal facilities [19,27,28]. IAEA and NEA OECD reports have argued for the safety of SNF and HLW disposal [29,30]. Many countries, including Russia, are already implementing programs to select sites for the construction of HLW disposal facilities [4,31,32]. Methods have been developed for partitioning of PUREX nitric acid waste into fractions [2,11,33,34,35]. The composition of the extraction mixture depends on the purpose of partitioning: For the extraction of Cs and Sr, CSEX and DDC processes are available; for REE and TPE—TRUEX, UREX, TRPO, TODGA, DIAMEX; for extraction of all actinides—GANEX; UNEX is used for the Cs + Sr + REE + TPE group with subsequent separation into Cs–Sr and REE–TPE fractions. Some of these techniques have been tested on actual liquid HLW, confirming high levels of technological readiness—TRL6 or higher [36]. The presence of stable mineral-like phases and environments where actinide migration is absent or very low [19] proves the possibility of effectively handling actinide by incorporation into matrices and burial [37,38]. Localization of radionuclides will be ensured by engineering barriers and host-rocks’ properties around a disposal facility (a mined repository or very deep borehole). Durable matrix (waste form) is the main engineering barrier of the HLW repository [39]. When choosing the matrix of the REE–MA fraction, it should be taken into account that lanthanides (La, Ce, Pr, Nd, Sm) dominate in its composition, and minor actinides (MA = Am, Cm) account for from 5 to 10 wt.% (Table 1) in the REE–MA mixture.

## 2. Requirements (Selection Criteria) for High-Level Waste Immobilization Matrices

The search for matrices for HLW fractions has been ongoing for several decades, and a number of criteria have been proposed [40]: (1) High waste loading (at least 20–35 wt.%) in order to minimize the volume and to maximize effective use of the repository; (2) simplicity, cost-effectiveness, and feasibility of manufacture; (3) resistance to radiation to exclude phase transformations over time, which reduce the stability of matrices; (4) corrosion resistance in water to prevent leaching of radionuclides, and their removal into the environment and the biosphere. A more detailed list is provided by references [41,42,43]: (1) High waste loading (up to 35 wt.%) to reduce waste volume in the geological repository. (2) Possibility of production under realizable conditions, using proven methods, minimizing the harmful effects on workers and reducing capital costs. (3) High resistance to radiation, including transmutation (transition into other elements), the effect of α-particles and recoil nuclei on the matrix, and the effect of radiation from the decay of fission products. (4) Flexibility, low sensitivity to changes in HLW composition, and the ability to include a mixture of radionuclides and other elements without formation of phases that degrade the properties of the matrix. (5) Stability in groundwater under disposal conditions, to reduce the leaching of long-lived actinide radionuclides and fission products. (6) Existence of natural analogues; testing of waste forms for time scales of thousands of years or more is not possible, but the presence of minerals with an age of hundreds of millions of years makes it possible to predict the behavior of the HLW matrix in a geological repository.

A similar set of criteria [44] takes into account the waste content in the matrix, the reality of industrial production, exclusion of a fission chain reaction, resistance to radiation, non-proliferation of fissile materials, and stability in underground waters. For actinide matrices, it is also necessary to exclude the possibility of spontaneous fission, for which it is proposed to introduce neutron absorbers REE or Hf in the matrix.

For vitrified HLW used in Russia, properties have been determined including uniformity of composition, maximal heat release, thermal and radiation resistance, leachability, mechanical strength, and thermo-physical parameters [45,46]. The main method of radionuclide release from the repository and their entry into the biosphere is leaching from the matrix by groundwater, therefore stability of solutions is among their most important properties.

Matrix properties can be divided into three groups: physical, chemical, and technological. The first group of parameters includes density, porosity, coefficients of linear and volume expansion, heat capacity, melting point (glass transition temperature for vitreous matrices), and thermal conductivity [45,47]. The chemical parameters consist of resistance to leaching, structural changes during radioactive decay, as well as crystal chemical parameters including the solubility of elements in glass, the capacity of the crystal structure, the valence and coordination of REE and actinide cations, the cation–oxygen distance, the strength of the cation field, and the type of elemental bonding. Technological properties are understood as physical properties that determine the possibility of obtaining a matrix on an industrial scale, for example, the melting temperature and the reaction rate during solid-state sintering. The physical and chemical parameters of matrices and their influence on the long-term behavior of solidified radioactive waste are summarized in Table 2.

Simulators (REEs) are typically used when studying MA (Am, Cm) matrices. Most often, Nd is used [48,49] due to the close ionic radii of Nd^3+^, Am^3+^, and Cm^3+^ in the same coordination [50,51] and the predominance of Nd in the REE-MA fraction (Table 1). Cerium can be used to simulate Pu, because it has oxidation states of 3+ and 4+ appropriate to Pu [44]. Isovalent and heterovalent isomorphism and exchanges involving vacancies play important roles in the immobilization of actinides in crystal structures [51,52,53,54,55,56,57]. The charge and size of cations affect the loading of matrices with waste, affectingsolubility in glass [58] and isomorphic capacity of the crystalline phases. When the content of elements is higher than this value, phases of REE and MA are formed, for example, REE and MA oxides, which is undesirable because of their higher solubility in water than that of target matrix phases.

The phenomena of morphotropy, polytypism and polysomatism are widely manifested in the HLW matrices. Titanates and zirconates of the REE_2_(Zr/Ti)_2_O_7_ composition are characterized by morphotropic transitions. As the ion radius decreases from light to heavy REE (from La^3+^ to Yb^3+^ and Y^3+^), the phase structure changes from perovskite to pyrochlore in titanates and from pyrochlore to fluorite in zirconates. For the former, the boundary lies between Nd^3+^ and Sm^3+^, for the latter it passes between Sm^3+^ and Gd^3+^. A dramatic change in the structure of the phases, by five orders of magnitude, affected the rate of REE leaching of titanate phases [59,60], from 1.5×10^−4^ g/(m^2^ × day) for Yb_2_Ti_2_O_7_ (pyrochlore) to 10 g/(m^2^ × day) in La_2_Ti_2_O_7_ (perovskite-like structure), but was found to have almost no effect on REE zirconates with pyrochlore or fluorite structures. Variations in the composition of pyrochlore REE^3+^_2_B^4+^_2_O_7_ (B = Ti, Sn, Zr) also influence initial rate of REE leaching: from 0.393 mmol/(m^2^ × day) for La_2_Sn_2_O_7_ to 0.007 mmol/(m × day) for Yb_2_Zr_2_O_7_ [61]. Morphotropic transitions can also affect the resistance of matrices to irradiation; decreasing for REEPO_4_ [62] when the structure of zircon (heavy REE phosphates) changed to monazite (light REE). Among REE titanates of (REE)_2_TiO_5_ composition, cubic phases are more resistant to radiation than compounds with hexagonal and rhombic structures [25].

Polytypism is associated with the existence of structures that differ only in their layer sequences, for example, when they are shifted or rotated with an increase in the period of the structure. The cell parameter in the direction of layer packing is a multiple of the distance between adjacent layers. Polytypes are usually designated by a combination of a number to show the number of layers in an elementary cell and a letter that indicates the symmetry [63]; cubic (C), hexagonal (H), rhombohedral (R), trigonal (T), orthorhombic (O), or monoclinic (M). The most widely known example of this phenomenon in the actinide matrices (waste forms) is due to five polytypes of zirconolite (2M, 4M, 3O, 3T, and 6T).

**Figure 1:**
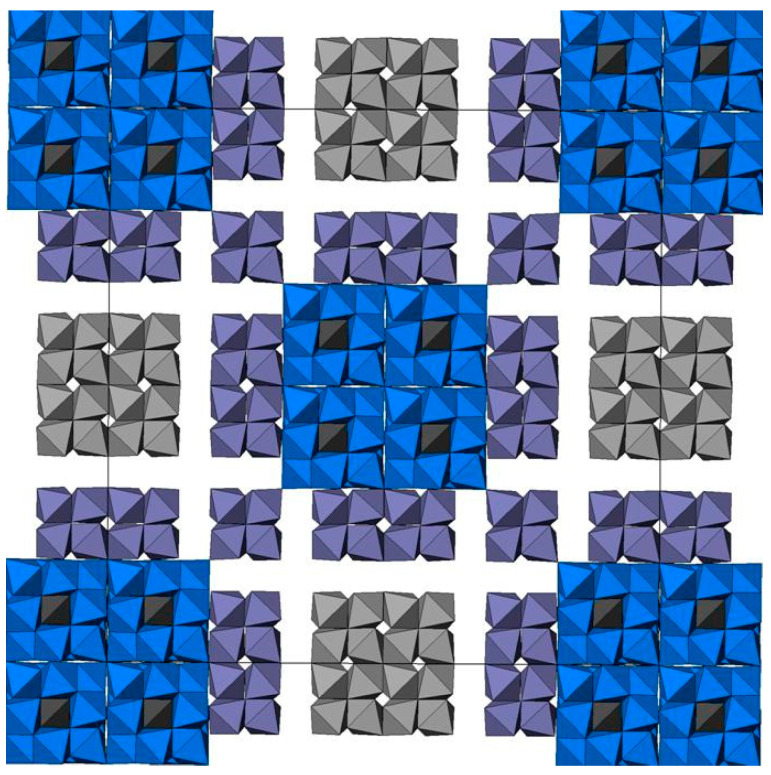
Combination of murataite 3C (blue)–pyrochlore (gray) modules and pyrochlore-type blocks (violet) in the structure of murataite 8C polysome.

The concept of polysomatism implies the presence of different blocks or modules in the structure of a compound [64,65]. The end members of polysomatic series have modules of the same type and composition. The structures of intermediate members (polysomes) consist of blocks of the end-member structures. They are present in HLW matrices in the form of murataite–pyrochlore polysomatic series [66,67,68], where pyrochlore and murataite 3C are the end members, and murataites 5C, 7C, and 8C are intermediate members (Figure 1) [68].

According to their different compositions, polytypes and polysomes are discernable in color in the backscattered electron (BSE) images of a scanning electron microscope, but establishing their structure and assigning them to a specific polytype (polysome) is a difficult task. Methods based on X-ray phase analysis and transmission electron microscopy have been used for this goal [66,67,68], not always possible in view of the small quantities of these phases in the samples. The aim of this article is to demonstrate the possibilities of electron backscatter diffraction (EBSD) in determining zirconolite polytypes and murataite polysomes in actinide matrices. First, we consider general information about the structural features of these groups of compounds.

## 3. Zirconolite and Murataite as Matrices for the Immobilization of Actinides

The choice of actinide matrix is largely determined by crystal–chemical parameters. At high temperatures, the most common oxidation states of actinides are Pu^3+/4+^, (Am, Cm)^3+^ and Np^4+^ [69]. REE elements of the REE-MA fraction (MA = Am, Cm) exist as trivalent ions, Ce can be partially in the form of Ce^4+^. To simulate actinides in matrices, lanthanides are used, and Ce, Th or U are introduced instead of Pu [47,70,71]. Due to close ionic radii [50], Ce^3+^ simulates Pu^3+^, while Nd^3+^ and Eu^3+^ simulate Cm^3+^ and Am^3+^. Monovalent Th^4+^ and Hf^4+^ are sometimes used to replace Np and Pu. Particular attention is paid here to studies using Nd, Sm, or La, which dominate among the REE fission products of SNF and in the composition of the REE-MA fraction of HLW. The average ionic radius of the REE–MA fraction is 1.11 Å, the same as for Nd^3+^, larger than that of Sm^3+^ (1.08 Å), but smaller than that of La^3+^ (1.16 Å). Therefore, Nd is the best simulant of the REE-MA fraction when studying the structure and waste loading of the matrices, the distribution of elements between phases, stability in water, and their main physical properties; density, heat capacity, thermal conductivity, and mechanical durability, which weakly depend on whether the simulant element is radioactive.

**Figure 2:**
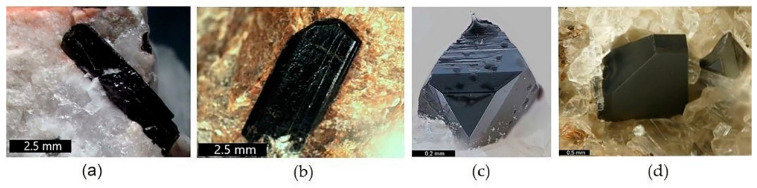
Natural zirconolites: (**a**,**b**) 3O (Vestfold og Telemark, Norway); and (**c**,**d**) 3T (Eifel Volcanic Fields, Germany; Fogo Volcano, Portugal).

For the immobilization of actinide containing nuclear waste, crystalline zirconolite and glass-crystalline materials with zirconolite have been proposed. They may be obtained by all known methods—sintering at atmospheric or elevated pressures, melting and crystallization, high-speed pulsed electric current sintering, and self-propagating high-temperature synthesis [57,72,73,74,75,76,77,78,79,80,81,82,83,84,85,86,87,88,89,90,91,92]. Zirconolite is stable in various natural conditions [22,24,25,75]; natural zirconolite is a rare mineral of terrestrial and lunar igneous rocks, and metasomatites. In nature, zirconolites from different localities have been found to contain UO_2_, ThO_2,_ or REE_2_O_3_ reaching 24, 22, or 32 wt%, respectively [23,25,93]. Due to the high conent of U and Th, zirconolite is often amorphous; this occurs at irradiation doses above 5 × 10^18^ α-decays/g [22]. In Figure 2, three minerals can be distinguished by composition: Zirconolite, monoclinic (polytype 2M), polymignite, orthorhombic (3O), zirkelite, hexagonal (3T) [94].

The above noted classification was approved by the Commission on New Minerals of the International Mineralogical Association [95]; hence, polymignite and zirkelite are no longer used. The 2M polytype is common in carbonatites, while the 3O and 3T polytypes are common in volcanics and metasomatites [96,97] with a content of REE^3+^, Th, Fe, and Nb.

**Figure 3:**
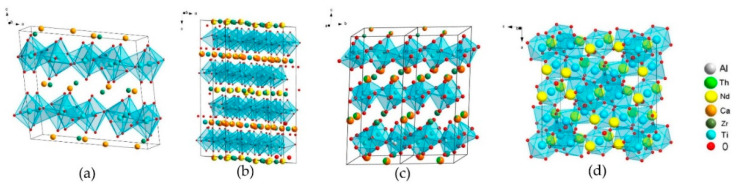
Structure of zirconolite polytypes (**a**) 2M, (**b**) 4M, (**c**) 3T, (**d**) pyrochlore Nd_2_(Ti,Zr)_2_O_7_.

Artificial zirconolite was observed for the first time in Synroc polyphase ceramics [72], an alternative to B–Si glass for HLW immobilization. In the first version, Synroc A, the (Zr,Ca,Ti)O_2_ oxide initially was determined, but X-ray microanalysis found that its formula corresponded to CaZrTi_2_O_7_ with an admixture of Al for charge balance when the Ca/Zr ratio varied. This allowed attribution of the phase found to an artificial analog of the mineral zirconolite, which later was confirmed by the X-ray diffraction data. In the all Synroc-type ceramics (B, C, D, E, F), zirconolite is the main host for actinides and REE [28,51,72,73,98]. It forms five polytypes—2M, 4M, 3O, 3T, and 6T, the figure is the number of layers of TiO_6_ octahedra, the letter is the symmetry of the lattice. The structure of the 2M polytype consists of trigonal and hexagonal rings of Ti-O octahedra, some of the Ti atoms are surrounded by 5 O^2−^ anions. These correspond to three positions of Ti atoms: Ti(1) and Ti(3) with a coordination number (cn) equal to VI (octahedrons), and Ti(2) with cn = V (bipyramids). The unit cell of zirconolite contains eight Ti(1) atoms, and four each of Ti(3) and Ti(2) atoms. Ca^2+^ (cn = VIII) and Zr^4+^ (cn = VII) are located between two networks of TiO_6_ octahedra. One structural module is formed by a pair of layers of TiO_6_ octahedra with interlayer cations. Its rotation through an angle multiple of 120° forms a cell of zirconolites 3T and 3O. With a change in the stacking sequence of Ca/Zr and Ti–O layers, other polytypes arise, and the structure of zirconolite 4M is a four-layer package of sheets of zirconolite 2M and pyrochlore (Figure 3).

The result is a doubling of the cell parameter along the *c* axis with preservation of monoclinic symmetry [99]. Variations in composition of the 2M phase are described by the formula CaZr_x_Ti_3–x_O_7_, “x” = 0.83–1.33. The replacement of Zr by Hf retains the 2M structure in CaHfTi_2_O_7_ and Ca_1−x_Nd_x_HfTi_2−x_Al_x_O_7_ (x = 0.01, 0.2) [100], which is due to the closeness of ionic radii of Zr^4+^ (0.78 Å) and Hf^4+^ (0.76 Å) (cn = VII).

Among the first works to note the effect of zirconolite composition on its structure were articles [51,74,101,102]. A description of all five zirconolite polytypes known so far is given in [103]. Their formation depends on the type of substitutions, charge and radius of cations, temperature, and oxidizing conditions [104]. Polytypes 2M and 4M are monoclinic (sp. gr. *C2/c*), 3O is orthorhombic (*Acam*), and 3T and 6T are hexagonal (*P3_1_21*). The most common polytypes in actinide matrices are 2M and 4M [104], less common are 3O [82] and 3T [70,105], there are no data for the 6T polytype. For phases of composition (Ca_1−x_^239^Pu_x_)Zr (Ti_2−2x_Fe_2x_)O_7_ (x = 0.1–0.7), the 2M polytype is stable up to “x” = 0.3, and 3T appears at “x” = 0.3 and 0.4. Replacing Pu with Ce increases the field of stability of the 2M polytype. Zirconolite 3T is often formed using thorium as a simulator. A reducing medium (5% H_2_/N_2_) is favorable for the formation of 3T zirconolite CaZr_1−x_Th_x_Ti_2_O_7_ at x ≥ 0.20 [104], Ca_0.8_Ti_1.35_Zr_1.3_Th_0.15_Al_0.4_O_7_ crystallizes in the same 3T type [106]. Fine intergrowth of 2M and 3T polytypes was established in glass ceramics obtained in the SiO_2_–Al_2_O_3_–CaO–ZrO_2_–TiO_2_–ThO_2_ system [80].

CaZr_1−x_(Ce/U/Th/Pu)_4+x_Ti_2_O_7_ (x = 0.1–0.6) phases are represented by zirconolite 2M, 4M and/or pyrochlore (Table 3), and their structures shown in Figure 3, with data from [104].

Information about zirconolite polytypes in glass ceramics is controversial. On the one hand [88,89], in B–Si glass ceramics, with an increase in the contents of CeO_2_ and Nd_2_O_3_ up to 15 wt%, transition of 2M polytype into 4M was observed. However, when studying glass ceramics with Ca_1−x_Zr_1−x_Nd_2x_Ti_2_O_7_, Ca_1−x_Nd_x_ZrTi_2−x_Al_x_O_7_, and CaZr_1−x_Ce_x_Ti_2_O_7_ (x = 0–0.5) phases, no transition of the 2M to 4M polytype was observed, in contrast to ceramics of the same composition [107]. In glass ceramics with REE and actinides, the 2M polytype is more stable due to the limited solubility of tri- and, especially, tetra-valent cations in the original glass [58,108] and low distribution coefficients of Nd^3+^, Ce^3+^, Th^4+^ between zirconolite and glass [80,109,110] during crystallization in the glass ceramic.

Compositions of natural and artificial zirconolite can be affected by substitutions [23,51,75], the main ones being (Ce,An)^4+^ → Zr^4+^; 2(Ln,An)^3+^ → Ca^2+^ + Zr^4+^; (Ln,An)^3+^ + (Al,Fe)^3+^ → Ca^2+^ + Ti^4+^; and more rarely: An^4+^ + (Fe,Co)^2+^ → Ca^2+^ + Ti^4+^; (Ce,An^4+^) + 2(Al,Fe,Cr)^3+^ → Ca^2+^ + 2Ti^4+^ (Ln are lanthanides, An are actinides). The replacement of 2M zirconolite by more complex 4M, 3O, or 3T polytypes has been observed with an increase in the concentration of tri- (La, Ce, Nd, Dy, Y, Am, Cm) and tetravalent ions (Ce, U, Np, Pu), replacing Ca^2+^ and Zr^4+^ [44,104,111,112,113]. An increase in the content of Nd^3+^ or (Ce/U/Th/Pu)^4+^ in the samples resulted in the zirconolite sequence structure 2M—3T—4M—pyrochlore [100,104].

In many early [28,72,73,98,114,115] and more recent [92,116,117] studies of matrices, the zirconolite polytype was not specified. There are several explanations for this and the first is that the similarity of properties [80] may make it unnecessary to identify the exact zirconolite polytype. There is no clear evidence that structural features somehow affect the resistance of zirconolite to radiation or corrosion in water [44]. It can only be argued that the polytypes have different capacities with respect to the actinide and REE-actinide fractions, i.e., maximum value for 4M, minimum for 2M, and intermediate for zirconolite 3O or 3T. With an actinide and REE content of up to 0.20–0.25 atoms, the zirconolite polytype 2M is stable [118,119]. The boundary between 2M and 4M polytypes for the Ca_1−x_Zr_1−x_Sm_2x_Ti_2_O_7_ solid solution passes at x = 0.35 [120].

Artificial zirconolite with REE and Pu is stable in alkaline and acidic solutions up to 500°C at 50 MPa, even after amorphization of the crystalline lattice [23,77,83,121,122,123,124].

**Figure 4:**
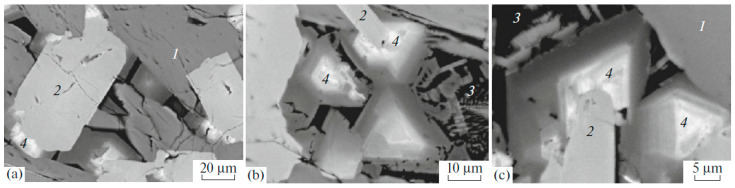
SEM images of the melted Synroc-type ceramic with model nuclear wastes: (1) hollandite, (2) zirconolite, (3) rutile and glass, (4) zoned murataite crystals overgrown on the zirconolite grains. (**a**) general view; (**b**,**c**) details with murataite–zirconolite intergrowths.

Murataite was first discovered in the Synroc matrix from defense waste obtained by sintering [125]. In the sample from the HLW simulator [66,67,126], obtained by melting and crystallization, it was formed from the melt last, growing on zirconolite grains (Figure 4) [127].

**Figure 5:**
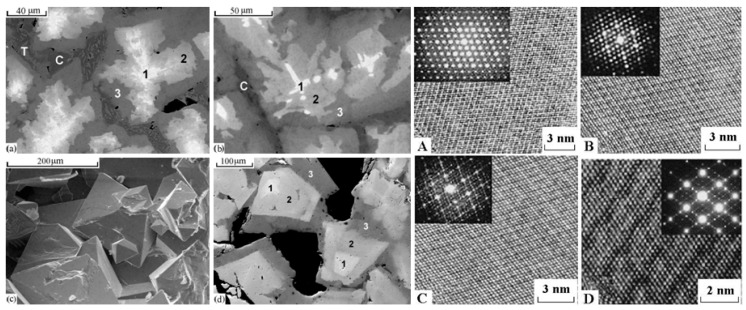
Left: SEM images of ceramics with 10% (**a**) UO_2_, (**b**) PuO_2_, (**c**,**d**) ThO_2_. (1) murataite 5C, (2) murataite 8C, (3) murataite 3C, (C) crichtonite, (T) pyrophanite; pores are black. Right: HRTEM micrographs of (**A**) pyrochlore, murataite (**B**) 3C, (**C**) 5C, (**D**) 8C taken with a high-resolution transmission electron microscope. Inserts: SAED patterns. Lines are atomic layers.

Such a close relationship between these two phases is due to the affinity of the structures both derived from the fluorite-type lattice. Murataite is this optimal host phase for tetravalent actinides (Th, U, Np, Pu); in this case it dominates in the matrix (Figure 5) [66,67].

In the nature, murataite has been found in only two locations, making it significantly rarer than zirconolite. Unlike zirconolite, murataite contains neither U nor Th, and of the rare earth elements contains only Y.

With an increase in the content of trivalent REE in the sample, perovskite-like phase has been observed, a less stable phase at elevated water solution temperatures, especially above 100 °C [128]. Synthetic murataites occur as phases 3C, 5C, 7C, and 8C (the number is the multiplicity of the cell parameter relative to the fluorite cell, C is the cubic symmetry of the lattice). They constitute the polysomatic series murataite 3 C—pyrochlore [68,129,130,131].

Murataites 5C and 8C are the most common in samples, whereas 3C is less common and 7C is very rare. All of them crystallize in the sp. gr. F-43m, so their X-ray diffraction patterns are similar. In melted ceramics, murataite forms zoned crystals with pyrochlore or murataite 5C at the center, and murataite 8C or 3C towards the edges (Figure 5). In samples obtained by sintering, murataite 5C and murataite 8C formed separate grains [132]. The pyrochlore module is responsible for actinide content, and the murataite block is responsible for corrosion products (Fe, Al, Mn). Although the concentrations of actinides decrease in the order pyrochlore–murataite 5C–8C–3C, while Fe and Al increase, the assignment of the phase to a specific polysome only by its composition may be incorrect.

Elucidation of the polytype (polysome) of phase requires examination by X-ray phase analysis with attention to weak reflections from a certain range of angles, or in a transmission electron microscope [82,100,103]. This problem is often complicated by the multiphase composition of matrices and the presence of several phases with similar structures—zirconolite, pyrochlore, and murataite. For simultaneous study of the composition and structure of phases, a combination of scanning electron microscopy (SEM) and electron backscatter diffraction (EBSD) can be applied [133,134]. The possibilities of this approach will later be shown using the example of samples containing zirconolite and murataite, but first we must present brief information about the electron backscatter diffraction method.

## 4. Characteristics of Electron Backscatter Diffraction Method

**Figure 6:**
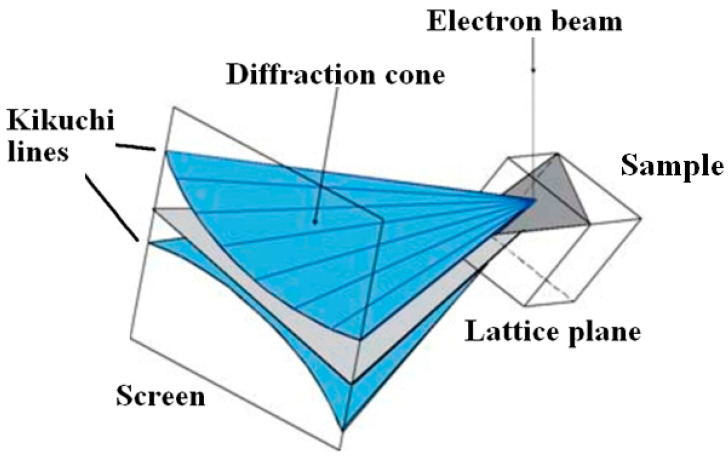
Obtaining of Kikuchi Lines in a scanning electron microscope by the EBSD method.

The EBSD method is based on the scattering of an incident electron beam in a sample, with the formation of a “point” source of electrons that coherently scatter and create a diffraction pattern which is recorded by a CCD detector. It presents a set of intersecting light stripes bounded by dark lines, called Kikuchi bands after the name of the scientist who first described this effect [135]. The method has been used in scanning electron microscopy since the 1970s [136,137]. To obtain EBSD patterns, the sample (Figure 6) is tilted at an angle of 70° which allows an increase in the proportion of electrons leaving the sample.

When the Bragg diffraction condition is met, two cone-shaped electron beams are formed for each family of planes, and fixed on the screen. The digital camera is positioned horizontally so that the screen is closer to the sample for a wider capture of the diffraction pattern. The width of the Kikuchi bands is proportional to the doubled Bragg reflection angle (2 theta) and inversely proportional to the interplanar spacing (d_hkl_). The angles between the Kikuchi bands are related to the angles of the crystallographic planes, and the points of their intersection correspond to the projections of the zone axes. EBSD pattern analysis makes it possible to determine the size and orientation of grains and their boundaries, to reveal the stress–strain state, the process of recrystallization, and the structure of phases.

In recent years, EBSD has been used in nuclear power engineering to analyze fuel based on UO_2_ or U-Mo and its claddings (Zr-Nb alloys) before and after irradiation in a reactor [138], and also the effect of annealing on the structure of materials [139,140]. In certain works [141,142] it is used as an auxiliary technique for studying the structure and phase composition of nuclear waste matrices. However, the possibilities of this method are much wider [133,134]. The purpose of this work is to study the structure of zirconolite and murataite in matrices with actinide simulators (Th, Nd) obtained by melting in air at 1500 °C in glassy carbon crucibles.

## 5. EBSD Study of Zirconolite Polytypes and Murataite Polysomes

### 5.1. Zirconolite—Murataite Matrix with Thorium (Sample “Th”)

**Figure 7:**
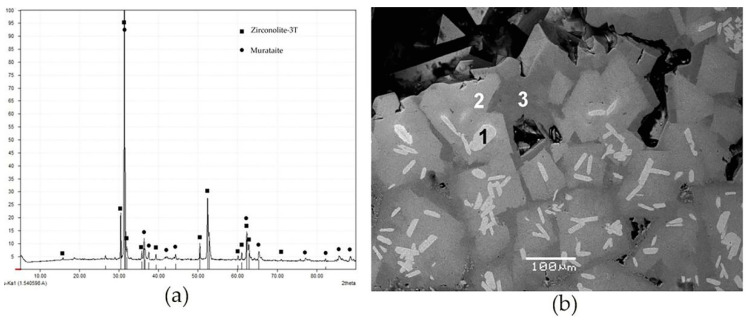
(**a**) X-ray powder diffraction pattern and (**b**) SEM image of sample “Th”: 1—zirconolite, 2—murataite—1, 3—murataite 2, blackp—ores. Markers on the XRD pattern show the peak positions only. The high-resolution diffraction pattern is given in Appendix A.

The composition of the sample was wt%: 50 TiO_2_, 10 CaO, 10 MnO, 5 Al_2_O_3_, 5 Fe_2_O_3_, 10 ZrO_2_, 10 ThO_2_. Powder diffraction pattern showed reflections of murataite (major phase) and zirconolite (Figure 7a). According to SEM/EDS data (Figure 7b), the main volume was occupied by gray grains surrounded by a dark mass. In the center of the grains are light elongated zirconolite crystals. The phase compositions are shown in Table 4; average values (wt%) as follows: 4.2 Al_2_O_3_, 10.5 CaO, 49.8 TiO_2_, 8.7 MnO, 2.8 Fe_2_O_3_, 11.4 ZrO_2_, 12.5 ThO_2_ (gray, murataite-1); and 8.8 Al_2_O_3_, 9.5 CaO, 54.7 TiO_2_, 11.0 MnO, 7.3 Fe_2_O_3_, 2.1 ZrO_2_, 6.6 ThO_2_ (dark, murataite-2). The difference in the color of the grains of phases 1 and 2 was caused by different contents of Th, Zr, Fe, and Al. Composition of zirconolite, wt%: 1.8 Al_2_O_3_, 10.2 CaO, 39.7 TiO_2_, 4.0 MnO, 1.4 Fe_2_O_3_, 29.5 ZrO_2_, 13.3 ThO_2_.

**Figure 8:**
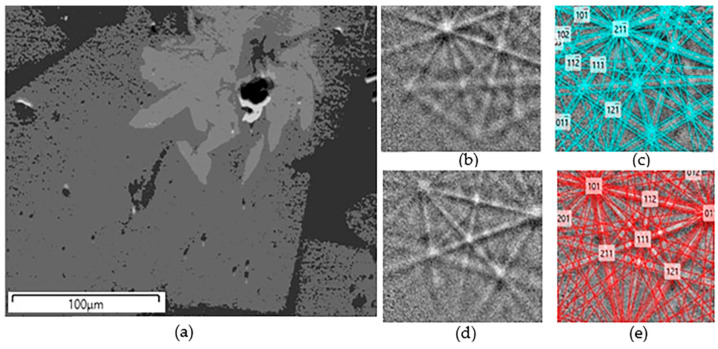
(**a**) SEM image of the analysed area, light—zirconolite, gray and dark-gray—murataite, and EBSD patterns of (**b**,**c**) zirconolite and (**d**,**e**) murataite, (**b**,**d**) before and (**c**,**e**) after indexing.

According to SEM/EDS analysis data, the phases were formed from the melt in the sequence zirconolite—murataite-1—murataite-2. The presence of two phases of murataite makes it important to identify their polysome and zirconolite polytypes. In the region of angles 2 theta ~30°, the peaks of zirconolite and murataite overlapped, and the low amount of zirconolite in the sample, ~10 vol.%, complicated the problem. The EBSD method served as the optimal technique for solving it. The EBSD pattern was obtained for one of the sections (Figure 8) for this purpose, with processing and indexing of the bands carried out automatically. Analysis of the EBSD patterns of zirconolite allowed its 3T polytype to be determined, and both varieties of murataite were found to be 8C polysomes. According to SEM/EDS data, the zirconolite formula was Ca_0.67_Th_0.19_Ti_1.84_Zr_0.89_Fe_0.07_Mn_0.21_Al_0.13_O_7_. The formulas for murataite calculated on 823 O^2−^ [68] corresponded to Ca_76.52_Th_19.4_Ti_254.73_Zr_37.86_Fe_14.54_Mn_50.35_Al_33.55_O_823_ for the central part (m-1) and Ca_65.04_Th_9.53_Ti_259.29_Zr_6.31_Fe_34.68_Mn_58.8_Al_65.04_O_823_ for marginal parts (m-2) of the zoned grains.

### 5.2. Pyrochlore—Zirconolite Ceramic with Neodymium (Sample “Nd”)

As mentioned earlier, Nd^3+^ serves as an analogue of Am^3+^ and Cm^3+^ as well as of the REE-MA fraction as a whole. Therefore, when searching for a matrix for the REE-MA fraction, neodymium titanates and zirconates are of great interest. In the NdO_1.5_—TiO_2_—ZrO_2_ system, the following phases are formed [143]: Nd_2_(Ti,Zr)_2_O_7_ (pyrochlore structure), Nd_2_Ti_2_O_7_ and Nd_2_Ti_4_O_11_ (perovskite-like structures), Nd_2_TiO_5_, and Nd_4_Ti_9_O_24_. Note that no information is given about the appearance of zirconolite in this system. These matrices were previously obtained by sintering or melting–crystallization [48,144,145,146].

**Figure 9:**
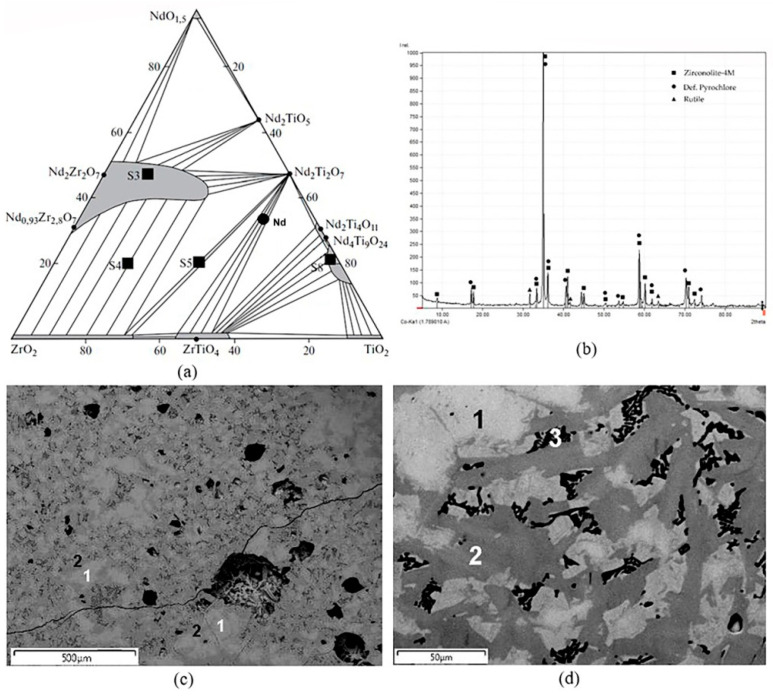
(**a**) Position of the “Nd” sample, (**b**) its X-ray diffraction pattern, and (**c,d**) SEM/EDS images: 1—pyrochlore, 2—zirconolite, 3—rutile (black) with fine pyrochlore inclusions. The scale bars on the SEM images are (**c**) 500 and (**d**) 50 µm. Markers on the XRD pattern show the peak positions only. The high-resolution diffraction pattern is given in Appendix B.

The sample “Nd” studied by the EBSD method was prepared by melting for 0.5 h at 1500 °C in a glassy carbon crucible. Its composition as well as spots of other samples (S3–S5, S8) are shown in Figure 9a. For ease of plotting, NdO_1.5_, TiO_2_, and ZrO_2_ are located at the vertices of the triangular diagram [143]. The composition, mol % 35 NdO_1.5_, 13 Nd_2_O_3_, 52 ZrO_2_ (or 21 Nd_2_O_3_, 16 ZrO_2_, 63 TiO_2_) corresponds to the association of pyrochlore Nd_2_(Zr,Ti)_2_O_7_ and srilankite ZrTiO_4_. Nevertheless, the X-ray diffraction pattern showed pyrochlore reflections and weak rutile peaks (Figure 9b). The SEM/EDS study established its composition of two neodymium titanium-zirconates as well as rutile (Figure 9, Table 5).

The grains of the light-colored phase had sections with shapes close to isometric. Taking into account the XRD data and the composition, it was determined as pyrochlore. The second phase (gray) composed elongated crystals surrounding light grains. Between them were black areas composed of rutile, with small inclusions of pyrochlore. The color of elongated crystals varied from gray to dark gray, reflecting the compositions (Table 5). The difference between isometric light grains and elongated gray grains was manifested in the content of TiO_2_ and, to a lesser extent, ZrO_2_ and Nd_2_O_3_. High, non-typical content of Nd_2_O_3_ in rutile is connected with the capture of fine grains of the light phase (pyrochlore) inside the aggregates of rutile grains. When calculating the phase formulas for two atoms Zr + Ti (pyrochlore) and four atoms Nd + Zr + Ti (zirconolite), a low value of the O^2−^ number in the pyrochlore formula and its high values in the zirconolite formula were obtained. Compared to the ideal formula A_2_B_2_O_6_O’, there was a deficiency of cations in the A position and an absence of the O’ anion in the formula of the pyrochlore. Defective pyrochlores were found in the samples S3–S5, S8 (Figure 9), obtained by melting–crystallization [144,147,148]. The difference between the formula of zirconolite and the ideal one with seven O^2−^ lies in the reduction of part of the Ti^4+^ to Ti^3+^ during the reaction of the melt within the carbon crucible: Ti^4+^O_2_ + C = Ti^3+^ + CO_2_. Taking this into account, a formula was calculated closer to the real values for zirconolite with the number of cations equal to four and seven O^2−^ anions (Table 5).

**Figure 10:**
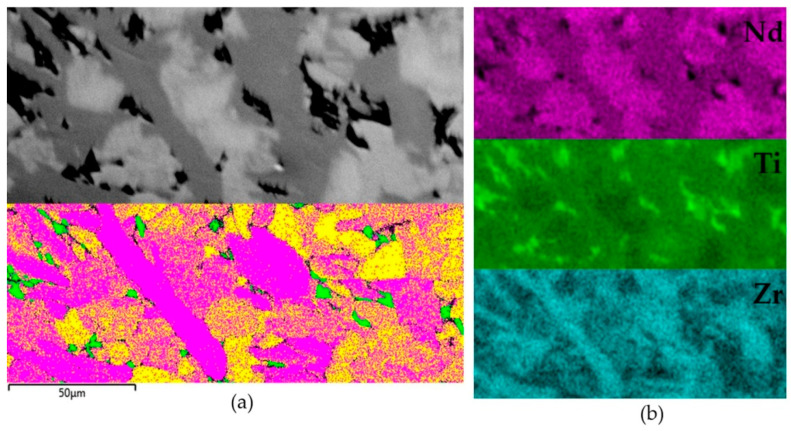
The structure of sample “Nd”: (**a**) top—BSE image (light—pyrochlore, gray—zirconolite, dark—rutile), bottom—EBSD map (yellow—pyrochlore, violet—zirconolite-4M, green—rutile); (**b**) distribution of Nd, Ti, Zr within the sample.

**Figure 11:**
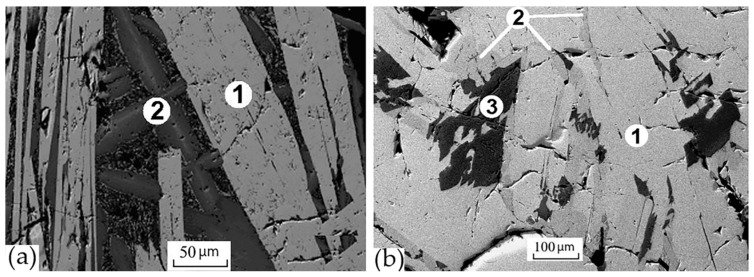
SEM images of the samples (**a**) MT: 1—monoclinic Nd_2_Ti_2_O_7_, 2—zirconolite; (**b**) RT: 1—orthorhombic Nd_4_Ti_9_O_24_, 2—zirconolite, 3—rutile.

To determine the structure of the light (pyrochlore) and gray (zirconolite) phases, the sample was studied by EBSD. As a result, the pyrochlore structure for the light phase was confirmed, and the best match for the EBSD patterns for the gray phase was found to be the 4M zirconolite polytype (Figure 10). These structural features contradict the data [143] suggesting the absence of zirconolite in the NdO_1.5_—TiO_2_—ZrO_2_ system. In most of the samples studied by us, obtained in this system by sintering or melting [148], for example, S3–S5, S8 (Figure 9), zirconolite was not found, and their phase composition mainly corresponded to the phase diagram (Table 6). Zirconolite was previously found only in two samples [48,144]. Their actual and calculated compositions (Table 7) differed in the presence of Al_2_O_3_ (impurity in the charge) and ZrO_2_ (Zr to initiate melting). As a result, in addition to monoclinic or rhombic Nd titanates, they were found to contain 10–30 vol.% of zirconolite (Figure 11).

Another reason for zirconolite’s appearance in the MT and RT samples (as in the sample “Nd”) is related to the reduction of part of Ti^4+^ to Ti^3+^ with the exchange: Ca^2+^ + Ti^4+^ = Nd^3+^ + (Al,Ti)^3+^. This explains the high O^2−^ value (7.29 and 7.37) and the different atomic amounts of Nd^3+^ and Al^3+^ in the formulas (Table 7). The reduction of Ti was probably caused by the high temperature of synthesis and the introduction of Zr (met.).

## 6. On the Simulators of Actinides and REE-Actinide Fraction in Nuclear Waste Matrices

Structural data obtained for a sample with stable elements and natural actinides (Th, U) is relevant to matrices with real radionuclides only if the simulator (REE, U, Th) is chosen correctly. For fractions of trivalent minor actinides (MA–Am, Cm) and REE–MA, Nd^3+^ serves as an optimal simulator due to the proximity of ionic radii in the corresponding coordination [48,107]. Their compounds have the same structure, for example monoclinic perovskite-like for Nd_2_Ti_2_O_7_ and Am_2_Ti_2_O_7_ [149], whilst REE titanates with a radius smaller than that of Nd^3+^ form a cubic pyrochlore structure. Hence, the use of Dy^3+^ as an simulator of Am^3+^ and other trivalent actinides is not quite correct [150]. A more complex picture can be observed when plutonium is replaced: Ce has often been used for this purpose due to the close ion radii in the Ce^3+^–Pu^3+^ and Ce^4+^–Pu^4+^ pairs. However, the stability fields of these ions do not coincide (Ce^4+^ is more easily reduced to Ce^3+^, the stability of Pu^4+^ is higher), and therefore no complete analogy is maintained between them. To simulate Pu, Ce^3+^ is recommended in a reducing environment, while Th^4+^ is recommended in an oxidizing one [104]. Sometimes ^VIII^Hf^4+^ (r = 0.83 Å) can serve as a Pu^4+^ simulator, but due to difference in ionic radii, this choice cannot be considered a good option.

## 7. Conclusions

Morphotropic transitions in actinide matrices composed of phosphates, titanates, and zirconates of rare earth elements resulted in a variety of structures of host phases. They also exhibited polytypism (zirconolite) and polysomatism (pyrochlore–murataite series). One of the important requirements for the matrix is a high waste content; therefore, the 4M, 3T, and 3O zirconolite polytypes and the 5C and 8C murataite polysomes are of interest. Their diagnostics by X-ray phase analysis can often be difficult due to the multiphase structures of matrices, with the presence of several phases at once structurally derived from the fluorite lattice (pyrochlore, zirconolite, murataite, cubic Zr oxide).

The structural types of phases in samples with simulators of actinide and REE-actinide fractions (Th, Nd) were determined using electron backscatter diffraction. The sample with Th contained zirconolite-3T of composition Ca_0.67_Th_0.19_Ti_1.84_Zr_0.89_Fe_0.07_Mn_0.21_Al_0.13_O_7_ and two phases—polysomes of murataite 8C: Ca_76.52_Th_19.4_Ti_254.73_Zr_37.86_Fe_14.54_Mn_50.35_Al_33.55_O_823_ and Ca_65.04_Th_9.53_Ti_259.29_Zr_6.31_Fe_34.68_Mn_58.8_Al_65.04_O_823_. The zirconolite in the sample with Nd was represented by the 4M polytype, pyrochlore is also present. The stability fields of zirconolite polytypes depend on the size of the cations that replace the main elements (Ca^2+^, Ti^4+^, and Zr^4+^). The replacement of Zr^4+^ by (Ce, U, Pu)^4+^ caused the transformation of the 2M polytype into 4M. Replacements of Ca^2+^–Zr^4+^ and Ca^2+^–Ti^4+^ with Pu^3+/4+^, REE^3+^, and small ions (Al, Cr, Fe, Ti)^3+^ stabilized polytypes 3O and 3T. We note the important role of pyrochlore in the structure of the 4M zirconolite polytype and the 8C polysome of murataite, which once again confirms the close relationship between the structures of these phases. In general, EBSD is an effective method for diagnosing polytypes and members of polysomatic series in HLW crystalline matrices.

## Figures and Tables

**Table 1:** (a) Composition (g/t) and (b) heat release (W/t) of SNF from light water reactors (LWR) depending on fuel burn and storage time [11].

Element	After 5 Years of Storage				After 30 Years of Storage			
	45 GW × d/t		60 GW × d/t		45 GW × d/t		60 GW × d/t	
	a	b	a	b	a	b	a	b
Gd, stable	150	0	310	0	180	0	346	0
Eu	190	60	260	90	170	8	230	12
Sm, stable	1060	0	1370	0	1120	0	1430	0
Pm	63	21	62	21	0	0	0	0
Ce	3210	10	4230	10	3210	0	4220	0
Pr	1540	114	2010	113	1540	0	2010	0
Nd, stable	5570	0	7310	0	5570	0	7310	0
La, stable	1670	0	2190	0	1670	0	2190	0
Σ REE	13,453	205	17,742	234	13,460	8	17,736	12
U	941,000	0.06	923,000	0.06	941,000	0.06	923,000	0.06
Pu	11,200	164	12,600	283	10,200	138	11,500	236
Np	570	0.01	780	0.02	570	0.01	780	0.02
Am	510	47	740	58	1380	146	1780	178
Cm	33	88	113	292	14	34	50	112
Am + Cm (MA)^3+^	543	135	853	350	1394	180	1830	290
REE/(REE + MA),%	96.2	60.3	95.4	40.1	90.6	4.3	90.7	4.0

REE, rare earth elements.

**Table 2:** Physical (nos. 1–12) and chemical (13–14) parameters of radionuclide matrices.

Nos.	Characteristic and Its Unit	Influence on the Properties of the HLW Matrix
1	Density, g/cm^3^	Amount of waste in the matrix and its volume
2	Poisson’s ratio	Mechanical strength and block stability
3	Young’s modulus, MPa	Mechanical strength and block stability
4	Compressive strength, MPa	Mechanical strength and block stability
5	Shear modulus, MPa	Mechanical strength and block stability
6	Radiation resistance, Gray	Exposure behavior on the decay of radionuclides
7	Thermal resistance, °C	Thermal behavior on the decay of radionuclides
8	Melting point, °C	Affects the matrix manufacturing technology
9	Glass transition temperature, °C	Thermal stability of glasses to crystallization
10	Expansion coefficient, °C^−1^	Thermal behavior on the decay of radionuclides
11	Specific thermal conductivity, W m^−1^ K^−1^	Heating during the decay of radionuclides
12	Heat capacity, J g^−1^ K^−1^	Heating during the decay of radionuclides
13	Solubility of waste, wt.%	Affects the amount of waste in the matrix
14	Leaching rates, g m^−2^ day^−1^	Ability of the matrix to retain radionuclides

**Table 3:** Phases in samples of bulk composition CaZr_1−x_(Ce/U/Th/Pu)_4+x_Ti_2_O_7_ at “x” from 0.1 to 0.6.

Cation	x = 0.10	x = 0.20	x = 0.30	x = 0.40	x = 0.50	x = 0.60
Ce^4+^	2M	2M + 4M	2M + 4M	2M + 4M	2M + 4M + P	4M + P
U^4+^	2M	2M + 4M	2M + 4M	4M + P	4M + P	4M + P
Th^4+^	2M + P	2M + P	2M + P	2M + P	2M + P	P
Pu^4+^	2M	2M + 4M	2M + 4M	4M + P	4M + P	P

2M, 4M—zirconolite polytypes, P—pyrochlore.

**Table 4:** Phase compositions of sample “Th” (∑ = 100 wt.%, SEM/EDS data).

Phase	Al	Ca	Ti	Mn	Fe	Zr	Th	O
Zirconolite	0.8	7.5	23.6	3.0	1.1	23.0	10.8	30.2
Zirconolite	0.9	7.1	24.6	3.5	1.3	19.6	12.8	30.2
Zirconolite	1.3	7.2	23.3	2.7	0.6	23.0	11.6	30.3
Murataite-1 ^1^	2.5	7.6	30.9	6.9	2.1	7.8	9.4	32.8
Murataite-1	2.0	7.8	29.2	6.8	1.9	10.0	10.2	32.1
Murataite-1	1.9	7.4	28.8	6.6	1.9	9.1	12.7	31.6
Murataite-2	4.6	7.0	33.1	8.2	4.5	2.6	5.2	34.8
Murataite-2	4.8	7.1	31.8	7.8	4.4	3.0	6.6	34.5
Murataite-2	4.7	6.4	32.8	8.7	4.8	2.0	5.9	34.7

^1^ Murataite 1 and 2—from the central and marginal parts of the zoned grains, respectively.

**Table 5:** Phase compositions averaged over three to five determinations (_c_—center, _e_—edge), wt%, and zirconolite formulae calculated for four cations (without Ti^3+^) or four cations and 7O^2-^ (accounting for Ti^3+^).

Phase	Ti	Zr	Nd	O	Only Ti^4+^ Suggested	Formulae of Zirconolite with Both Ti^3+^ and Ti^4+^
Pyrochlore	16.5	13.1	46.9	23.5	Nd_1.35_Zr_0.59_Ti_1.41_O_6.02_	Nd_1.35_Zr_0.59_Ti_1.41_O_6.02_
Zirconolite _c_	21.5	14.6	38.1	25.8	Nd_1.22_Zr_0.74_Ti_2.04_O_7.40_	Nd_1.22_Zr_0.74_Ti^4+^_1.26_Ti^3+^_0.78_O_7_
Zirconolite _e_	23.5	11.8	38.5	26.2	Nd_1.20_Zr_0.60_Ti_2.20_O_7.40_	Nd_1.20_Zr_0.60_Ti^4+^_1.40_Ti^3+^_0.80_O_7_
Rutile	46.8	7.0	10.7	35.5	Ti_0.86_Zr_0.07_Nd_0.07_O_1.97_	Ti_0.86_Zr_0.07_Nd_0.07_O_1.97_

**Table 6:** Chemical (mol.%) and phase compositions of samples: (a) expected [143]; (b) actual phases present according to XRD and SEM/EDS studies [148].

Sample	NdO_1.5_	ZrO_2_	TiO_2_	Phase Composition: Expected (a) and Real (b)
S3	50	37.5	12.5	P^a^/P^b^
S4	20	60	20	P—B/B—O—B
S5	20	40	40	B—TN—1/B—P—TZ
S8	23	0.02	75	TN—2/TN—2—R ^1^

^1^ P—pyrochlore Nd_2–x_(Ti, Zr)_2_O_7–1.5x_, B—monoclinic baddeleyite ZrO_2_, O—cubic oxide (Zr, Nd)O_2–x_, TN-1—Nd_2_Ti_2_O_7_, TZ—ZrTiO_4_, TN-2—Nd_4_Ti_9_O_24_, R—rutile (Ti, Zr)O_2_.

**Table 7:** (1) Calculated and (2) actual compositions of the MT and RT samples and their phases—monoclinic (m) and orthorhombic (o) Nd titanates and zirconolite polytype (z-3O).

Oxide/Ion	MT				RT			
	1	2	m	z-3O	1	2	o	z-3O
Al_2_O_3_	-	0.8	n.d. ^1^	3.1	-	2.2	n.d.	6.7
TiO_2_	32.9	28.5	31.4	33.1	52.5	48.5	50.5	37.6
ZrO_2_	-	6.2	0.8	25.2	-	5.0	1.6	19.6
Nd_2_O_3_	67.1	64.5	67.8	38.5	47.5	44.3	47.9	36.1
Al^3+^	-		n.d.	0.27	-		n.d.	0.54
Ti^4+^	2.00		1.96	1.82	9.00		8.82	1.93
Zr^4+^	-		0.03	0.90	-		0.18	0.65
Nd^3+^	2.00		2.01	1.01	4.00		4.00	0.88
O^2-^	7.00		6.99	7.37	24.00		24.00	7.29

^1^ n.d.—not detected. The target phases were monoclinic Nd_2_Ti_2_O_7_ (sample MT) and orthorhombic Nd_4_Ti_9_O_24_ (sample RT).

**Figure A1:**
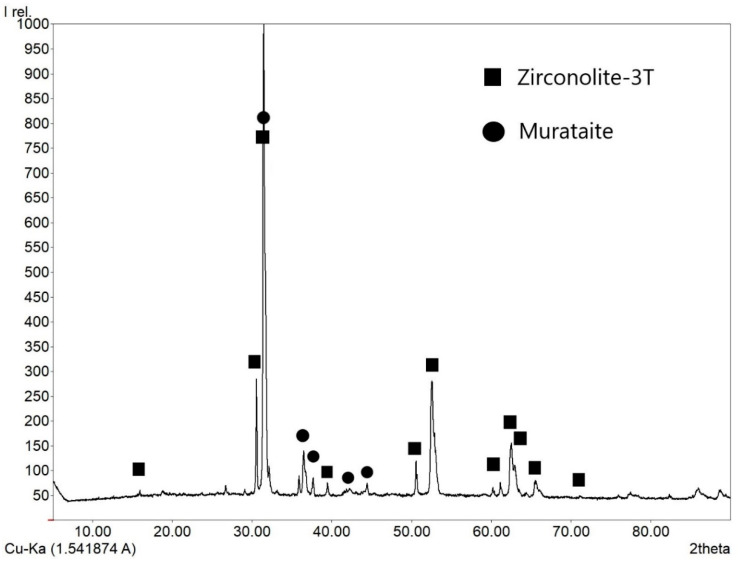
X-ray diffraction pattern of the sample “Th”.

**Figure A2:**
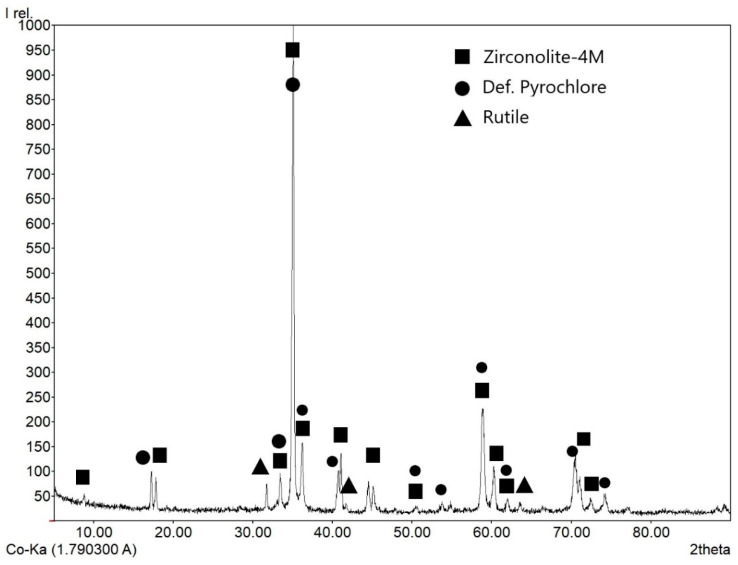
X-ray diffraction pattern of the sample “Nd”.

## Data Availability

All data are available within the paper.
